# Eye Movements Detect Differential Change after Participation in Male Collegiate Collision versus Non-Collision Sports

**DOI:** 10.1089/neur.2021.0030

**Published:** 2021-10-07

**Authors:** Virginia T. Gallagher, Prianka Murthy, Jane Stocks, Brian Vesci, Jeffrey Mjaanes, Yufen Chen, Hans C. Breiter, Cynthia LaBella, Amy A. Herrold, James L. Reilly

**Affiliations:** ^1^ Department of Neurology, UVA Health, Charlottesville, Virginia, USA.; ^2^ Department of Psychiatry and Behavioral Sciences, Northwestern University Feinberg School of Medicine, Chicago, Illinois, USA.; ^3^ Department of Sports Medicine, Northwestern University, Evanston, Illinois, USA.; ^4^ Center for Translational Imaging, Northwestern University, Evanston, Illinois, USA.; ^5^ Division of Orthopedics and Sports Medicine, Ann & Robert H Lurie Children's Hospital of Chicago, Chicago, Illinois, USA.

**Keywords:** collision sports, eye tracking, oculomotor, repetitive head trauma, subconcussive

## Abstract

Although neuroimaging studies of collision (COLL) sport athletes demonstrate alterations in brain structure and function from pre- to post-season, reliable tools to detect behavioral/cognitive change relevant to functional networks associated with participation in collision sports are lacking. This study evaluated the use of eye-movement testing to detect change in cognitive and sensorimotor processing among male club collegiate athletes after one season of participation in collision sports of variable exposure. We predicted that COLL (High Dose [hockey], *n* = 8; Low Dose [rugby], *n* = 9) would demonstrate longer reaction times (antisaccade and memory-guided saccade [MGS] latencies), increased inhibitory errors (antisaccade error rate), and poorer spatial working memory (MGS spatial accuracy) at post-season, relative to pre-season, whereas non-collision collegiate athletes (NON-COLL; *n* = 17) would remain stable. We also predicted that whereas eye-movement performance would detect pre- to post-season change, ImPACT (Immediate Post-Concussion Assessment and Cognitive Test) performance would remain stable. Our data showed that NON-COLL had shorter (improved performance) post- versus pre-season antisaccade and MGS latencies, whereas COLL groups showed stable, longer, or attenuated reduction in latency (*p*s ≤ 0.001). Groups did not differ in antisaccade error rate. On the MGS task, NON-COLL demonstrated improved spatial accuracy over time, whereas COLL groups showed reduced spatial accuracy (*p* < 0.05, uncorrected). No differential change was observed on ImPACT. This study provides preliminary evidence for eye-movement testing as a sensitive marker of subtle changes in attentional control and working memory resulting from participation in sports with varying levels of subconcussive exposure.

## Introduction

Participation in collision sports (e.g., American football, ice hockey, rugby, and soccer) increases a person's exposure to head impacts, which occur when a biomechanical force is imparted on the brain because of a blow to the head or body.^1,2^ A head impact may result in a concussion, in which clinical signs and symptoms are present, such as loss of consciousness, confusion, and amnesia.^2^ More frequently, however, head impacts fall into the category of subconcussive injuries in which no apparent clinical signs or symptoms are present, but pathophysiological changes in the brain affecting vasculature and white matter may occur and may have cumulative adverse effects over time (although this remains controversial given the infancy of the literature).^1,3–6^ Specifically, chronic exposure to repetitive head impacts, including concussive and subconcussive events, may be associated with increased risk for long-term neurocognitive and -psychiatric outcomes, including chronic traumatic encephalopathy, Alzheimer's disease, major depressive disorder, and deficits in executive functions, language, and memory.^4,7–9^ Subconcussive impacts are particularly concerning in adolescents because neural circuits critical for higher-order cognitive functions, including attentional control and working memory, develop throughout adolescence and into early adulthood.^10,11^

Neuroimaging studies assessing subconcussive events over the course of an athletic season have demonstrated longitudinal alterations in white matter structure and functional activation among high school and college athletes.^12–19^ Although neuroimaging methods have detected change associated with subconcussive injuries, it is not financially feasible for tracking change associated with exposure to subconcussive impacts on a large scale, and, more important, structural neuroimaging does not provide information regarding potential cognitive or behavioral consequences associated with cumulative subconcussive impact exposure.

Studies directly examining cognitive effects of subconcussive injury associated with collision athletics participation have yielded mixed findings, with some reporting pre- to post-season decline in processing speed, attention, and memory^19–22^ and others observing no change over the season.^23–26^ Given that cognitive change is observed variably in the short term, but more consistently in the long term,^9^ it is possible the cognitive measures currently used to evaluate possible subtle cognitive change associated with subconcussive impacts lack adequate sensitivity. Recently, eye-movement testing has emerged as a promising tool for detecting neurocognitive and sensorimotor change after concussive^27–29^ and subconcussive head trauma among female athletes.^30^ Eye-movement testing provides an assay to evaluate sensorimotor processing, speed of attentional shifting, executive control, inhibition, and working memory, which are domains often affected by head injury.^31^ Further, eye-movement testing is ideally suited to detect effects of brain trauma given that most aspects of classic saccadic eye-movement task performance have been localized to specific neuroanatomical areas and networks.^32^

The primary aim of this study was to evaluate whether eye-movement testing detects change in sensorimotor and cognitive processing after male collegiate athletes' participation in collision sports. We specifically focused on ice hockey and rugby, which have elevated rates of subclinical head trauma exposure.^33–35^ We compared the longitudinal eye-movement performance of male, collegiate club collision sport athletes (COLL) to male, collegiate club non-collision sport athletes (NON-COLL). Several classic saccadic eye-movement tasks were used in this study: prosaccade, antisaccade, and the memory-guided saccade (MGS) tasks (see Gallagher and colleagues for further task information).^30^ Given that saccade tasks assessing executive functions (antisaccade and MGS) were more sensitive to the effects of contact sport participation among female collegiate athletes relative to the less cognitively demanding prosaccade task,^30^ we expected that the antisaccade and MGS tasks would be more sensitive to the effects of collision sport participation among male athletes, relative to the reflexive visual attention prosaccade task.

In the main analyses of interest, we hypothesized that collision sport athletes would demonstrate the following changes (reflecting reduced executive attentional control and working memory ability) at post-season, relative to pre-season, whereas non-collision sport athletes would demonstrate stability over time: 1) longer antisaccade latency (reaction time); 2) increased antisaccade error rate (inhibitory errors); 3) longer MGS latency (reaction time); and 4) poorer spatial accuracy on the MGS task (spatial working memory).

We also explored whether collision sport athletes would demonstrate longer prosaccade latency, gain (overshooting), prolonged duration, and slower velocity at post-season, relative to pre-season, whereas we expected non-collision athletes to remain stable over time. Given that ImPACT (Immediate Post-Concussion Assessment and Cognitive Test) is the most commonly used tool in the literature to assess cognitive change as the result of exposure to subconcussive impacts, we also compared pre- to post-season change in cognitive abilities as measured by ImPACT.^36^ We hypothesized that among collision sport athletes, pre- to post-season change would not be observed on ImPACT whereas it would be observed on eye-movement testing.

## Methods

### Participants and recruitment

Male university club athletes between ages 18 and 25 years who planned to participate in at least 85% of team practices and games were recruited. Exclusion criteria were factors known to influence eye-movement performance such as a lifetime diagnosis of a psychotic disorder,^37^ a first-degree relative with a psychotic disorder,^38^ a history of a seizure disorder,^39^ a concussion within the previous 6 months, or lifetime history of moderate-to-severe head injury.^40^ Because of unforeseen changes in schedule and participation, male club ice hockey players were assigned to the COLL-High Dose group, and male club rugby players were assigned to the COLL-Low Dose group (see *Schedule of activities* section below). Male club crew, cross-country, swimming (divers excluded), and triathlon athletes were assigned to the NON-COLL group and all denied concurrent participation in intermural or club collision sports. All participants completed an in-person informed consent and were compensated at $20 per hour for their time. This study was approved by the university's institutional review board.

### Procedure

#### Schedule of activities

Athletes were assessed at two time points (pre- and post-season). For COLL, the pre-season visit was completed before the commencement of pre-season training and was scheduled to maximize the time since the end of the previous competitive season. Pre-season visit procedures for NON-COLL athletes occurred shortly after the start of the regular season competition because of the availability of athletes and research equipment.

Because of unforeseen circumstances, the rugby team's season unexpectedly ended halfway through the season, resulting in decreased playing time than expected (lower dose of exposure) and increased duration between the last game and the post-season assessment (mean = 19.00 days, standard deviation [SD] = 5.07). Post-season visits for COLL-High Dose took place an average of 11.38 days (SD = 14.00) after the most recent competitive match. NON-COLL athletes were scheduled for post-season visits so that the mean duration between pre- and post-season would be the same for both groups. At each visit, participants completed eye-movement testing, questionnaires, and ImPACT testing. Athletes were instructed not to use caffeine, stimulants, or nicotine within 1 h preceding visits to reduce the potential effect of those confounding factors on eye-movement performance.^41^

#### Eye-movement testing

Eye movements were measured in a windowless room using the Eyelink1000 Plus system (SR Research Ltd, Kanata, Ontario, Canada), a state-of-the-art infrared video-recording system that permits for high-resolution (1000 Hz; microsaccade resolution of 0.05 degrees) recording of eye position. Primary eye-movement measurements of interest on the prosaccade task are latency, gain, accuracy, duration, and peak velocity, as detailed in past work.^30^ On the antisaccade task, the primary eye-movement measurements of interest are primary saccade latency and antisaccade error rate (error trials/total trials), a measure of executive inhibition and cognitive control. On the MGS task, the primary eye-movement measurements of interest are primary saccade latency, primary saccade accuracy, and resting position accuracy. See Gallagher and colleagues for further details.^30^

#### Immediate Post-Concussion Assessment and Cognitive Test

ImPACT is a 25-min computerized cognitive battery designed for ages 12–59, delivered by a secure Web portal.^42^ The test consists of six subtests that yield four normed composite scores: verbal memory, visual memory, visual-motor speed, and reaction time. Test version 3.4.0 was used in this study and was administered by a trained ImPACT administrator in group format in a computer laboratory for the majority of participants; because of scheduling issues, 3 participants completed ImPACT individually in a clinic room, administered by a trained ImPACT administrator, at both pre- and post-season.

#### Questionnaires

Before in-person study assessments, all participants completed a comprehensive questionnaire regarding demographic, health history (including concussion history), recent athletic participation information, recent substance use (30-day Timeline Followback),^43–45^ educational background, and parental education and occupational history (to yield a total socioeconomic status score).^46^

In addition, participants completed the following questionnaires online (within 24 h of eye-movement testing) at both visits to assess the influence of physical, cognitive, and emotional symptoms on eye-movement performance. The Post-Concussion Symptom Scale (PCSS), a commonly used measure in clinical and research domains, was used to assess presence and severity of physical, cognitive, and emotional symptoms that occur in both concussed and healthy populations.^47^ The PCSS symptom severity score (range, 0–132) was used in analyses. The Beck Depression Inventory-II (BDI-II) is a 21-item self-report questionnaire that was used to assess the characteristic attitudes and symptoms of depression.^48,49^ The State-Trait Anxiety Inventory (STAI), a commonly used 40-item measure of trait and state anxiety, was used to differentiate situational versus dispositional anxiety.^50^ The Perceived Stress Scale (PSS) is a 10-item questionnaire that was used to assess baseline levels of one's ability to handle stress.^51,52^

#### Participation and exposure measurement

To grossly estimate exposure to collision sport participation, team captains for COLL teams recorded participant practice and game attendance; players were marked as “not present” if they attended but did not participate in a practice or game. Total minutes of attendance was calculated by summing the athletes' total number of practices/games and multiplying that sum by practice/game length, respectively. COLL athletes also self-reported the date of the most recent competition they participated in, and COLL and NON-COLL athletes self-reported the dates of the most recent practices they participated in; this information was cross-referenced with attendance logs provided by COLL team captains; attendance logs were not available for NON-COLL athletes.

### Data analyses

See Gallagher and colleagues for eye-movement data scoring and cleaning procedures.^30^

#### Statistical analyses

Statistical analyses were performed using IBM SPSS Statistics for Windows (Version 25.0; IBM Corp., Armonk, NY).^53^ Descriptive analyses were conducted to evaluate group differences in demographic and clinical history information; specific tests included one-way analyses of variance and chi-square tests. General linear mixed modeling of trial-wise data was used to evaluate group differences on eye-movement measures of interest within task. See the Supplementary Materials for further details on eye-movement statistical analyses. Pre- to post-season change on ImPACT performance was conducted using a repeated-measures multi-variate analysis of variance. Alpha thresholds for the five main hypotheses tests (four eye movement, one ImPACT) were corrected for multiple comparisons (Bonferroni corrected, *p* = 0.01 [0.05/5]). Effect sizes (d_ppc2_) were calculated for significant main hypotheses testing results; effect sizes reflect the difference in mean pre- to post-season change in eye-movement performance (averaged across conditions) between the COLL-High Dose versus NON-COLL group or between the COLL-Low Dose versus the NON-COLL group, divided by the pooled pre-season SD.^54^

## Results

### Enrollment, demographic, mood, and substance-use data

The final sample included 8 COLL-High Dose, 9 COLL-Low Dose, and 17 NON-COLL athletes. The NON-COLL group consisted of 9 crew, 3 swimming, 2 triathlon, 2 cross-country, and 1 track athletes. One COLL-High Dose athlete sustained a concussion during the season; the injury was sustained 108 days earlier, and he was cleared for full return to play 87 days before the post-season assessment. All statistical analyses were first completed with the concussed COLL-High Dose player included and then repeated with his data excluded; results were consistent. Therefore, this subject was retained in the sample.

Pre-season assessments among hockey players were conducted an average of 30.89 weeks (SD = 3.51, range = 22.28–33.29) after the athletes' most recent competitive game; pre-season assessments among rugby players were conducted an average of 9.86 weeks (SD = 0.96, range = 8.71–11.29) after the athletes' most recent competitive game. There were no significant group differences in demographic or health history information; see Table 1. There were significant group differences in pre-season BDI-II total scores and duration between visits, but neither of these factors were associated with eye-movement performance at baseline or change on eye-movement testing from pre- to post-season; see Table 2. There was also no visit (pre- vs. post-season) or group-by-visit effects on mood or symptom ratings (BDI-II, PCSS, STAI, or PSS). Groups differed on mean days since most recent practice participation (COLL-High Dose vs. COLL-Low Dose vs. NON-COLL) and total minutes of game attendance (COLL-High Dose vs. COLL-Low Dose); see Table 2.

**Table 1.** Sample Demographic and Health History Information

	Group		
	Collision-High Dose	Collision-Low Dose	Non-collision
	Hockey (*n* = 8)	Rugby (*n* = 9)	(*n* = 17)
Age, M (SD)	19.38 (0.92)	19.78 (1.48)	19.47 (0.94)
Race (% Caucasian)	88%	56%	82%
Ethnicity (% Hispanic or Latino)	0%	22%	6%
Socioeconomic status (Hollingshead four-factor, M [SD])	162.88 (18.52)	152.29 (20.70)	131.64 (38.84)
Years of education, M (SD)	13.39 (0.92)	13.11 (1.27)	13.53 (0.94)
Hx of anxiety or depression	13%	22%	6%
Hx of learning disability or ADHD	0%	0%	3%
Hx of one or more previous concussions	38%	33%	24%

Data presented as mean (standard deviation), where indicated, or percentage (%) of group. Hx = self-reported history. Years of education reflects highest year of education completed (13 years = completed freshmen year of college). No significant group differences detected.

ADHD, attention-deficit hyperactivity disorder.

**Table 2.** Mood, Substance Use, and Participation Data

	Group			
	Collision-High Dose	Collision-Low Dose	Non-collision	
	Hockey (*n* = 8)	Rugby (*n* = 9)	(*n* = 17)	*p* value
**Visit 1 information**				
BDI-II total	2.50 (1.93)	9.22 (9.43)	3.94 (3.61)	0.035
PCSS total	8.38 (8.78)	14.11 (12.21)	12.00 (12.18)	ns
STAI state total	28.63 (3.81)	37.56 (15.15)	35.00 (9.70)	ns
STAI trait total	28.13 (3.56)	40.89 (15.83)	35.47 (9.10)	ns
Perceived stress total	8.88 (3.72)	14.89 (9.27)	12.88 (5.53)	ns
Total days alcohol consumption in previous 30 days	8.88 (6.62)	8.56 (7.80)	5.00 (5.43)	ns
Total servings of alcohol previous 30 days	36.00 (23.82)	35.44 (40.73)	31.00 (37.34)	ns
Average servings alcohol/consumption day	4.30 (0.89)	4.17 (1.33)	5.50 (2.50)	ns
**Visit 2 information**				
BDI-II	2.75 (2.44)	10.44 (12.84)	4.50 (4.73)	ns
PCSS	11.50 (11.65)	22.56 (24.26)	13.59 (15.95)	ns
STAI state total	28.88 (6.38)	40.44 (17.02)	36.24 (11.49)	ns
STAI trait total	32.25 (8.26)	41.33 (18.11)	35.82 (11.71)	ns
Perceived stress total	10.88 (3.00)	17.44 (8.85)	14.65 (6.54)	ns
Total days alcohol consumption in previous 30 days	8.25 (5.29)	8.78 (7.88)	4.00 (4.29)	ns
Total servings of alcohol previous 30 days	41.38 (32.53)	33.72 (29.97)	23.41 (34. 26)	ns
Average servings alcohol/consumption day	4.93 (1.45)	3.75 (2.26)	4.70 (3.25)	ns
Weeks between pre- and post-season visit	20.29 (0.22)	17.32 (0.38)	18.55 (1.44)	<0.001
Participation and exposure information				
Days since most recent practice participation: post-season visit	6.38 (7.11)	19.00 (7.48)	3.35 (5.53)	<0.001
Days since most recent competition participation: post-season visit	11.38 (14.00)	19.00 (5.07)	—	ns
Total minutes of practice attendance	1766.25 (464.77)	1750.00 (607.73)	—	ns
Total minutes of competition attendance	1140.00 (525.03)	89.89 (75.15)	—	<0.001

Data presented as mean (standard deviation), unless otherwise indicated. ns = no significant differences among or between groups.

BDI-II, Beck Depression Inventory-II; PCSS, Post-Concussion Symptom Scale; STAI, State-Trait Anxiety Inventory.

### Eye movement

See Supplementary Tables S1–S3 for descriptive eye-movement data among groups. See Table 3 for general linear mixed-model results with significant visit-by-group-by-condition and/or visit-by-group interaction effects. Only main effects of interest (visit-by-group-by-condition and visit-by-group) are reported in the Results section. Given the high correlation between saccade gain and accuracy (*r*_(5582)_ = 0.848, *p* < 0.001), only the results of gain analyses are reported to reduce redundancy.

**Table 3.** Significant Group (COLL-High Dose vs. COLL-Low Dose vs. NON-COLL) General Linear Mixed-Model Results

Task	Measure	Predictor	*df*_*numerator*_	*df*_*denominator*_	*F*	*p* value
Prosaccade	Latency	Visit	1	5482	0.823	0.364
		Condition	2		840.458	<0.001
		Group	2		0.173	0.841
		Visit^*^Group	2		1.862	0.155
		Visit^*^Group^*^Condition	10		7.616	<0.001
	Peak velocity	Visit	1	2014	5.570	0.018
		Group	2		0.777	0.46
		Visit^*^Group	2		5.208	0.006
Antisaccade	Latency	Visit	1	4599	26.084	<0.001
		Condition	2		545.363	<0.001
		Group	2		0.317	0.729
		Visit^*^Group	2		6.645	0.001
		Visit^*^Group^*^Condition	10		3.970	<0.001
Memory-guided saccade	Latency	Visit	1	3893	0.411	0.522
		Condition	3		470.716	<0.001
		Group	2		3.958	0.019
		Visit^*^Group	2		20.614	<0.001
		Visit^*^Group^*^Condition	15		3.508	<0.001
	Primary saccade accuracy	Visit	1	3959	0.002	0.962
		Condition	3		25.059	<0.001
		Group	2		0.323	0.724
		Visit^*^Group	2		3.061	0.047
		Visit^*^Group^*^Condition	15		1.423	0.127
	Resting fixation accuracy	Condition	3	4047	5.694	0.001
		Visit^*^Group	5		2.397	0.035

Only includes analyses with significant visit-by-group or visit-by-group-by-condition interaction effects. Asterisk (“^*^”) signifies a two- or three-way interaction effect.

COLL, collision; NON-COLL, non-collision.

#### Prosaccade task

**FIG. 1.**
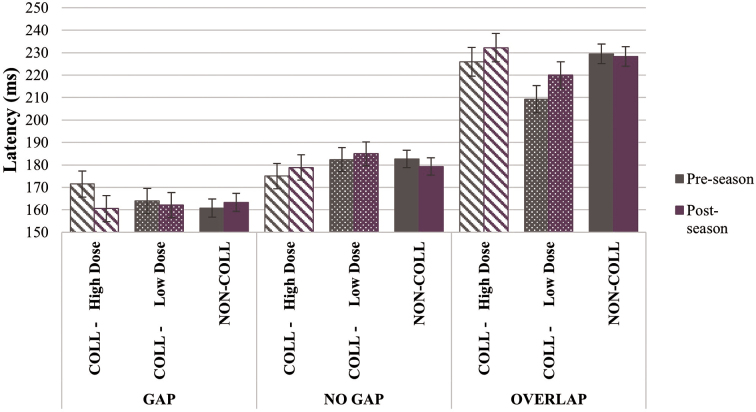
Prosaccade latency by visit, group, and condition. Compared to pre-season performance, *COLL* groups demonstrated longer latency on no gap and overlap, but shorter latency on gap at post-season; *NON-COLL* demonstrated relatively stable latency across conditions from pre- to post-season (visit-by-group-by-condition interaction effect, *p* < 0.001). COLL, collision; NON-COLL, non-collision.

On the reflexive, prosaccade task, there was a visit-by-group-by-condition interaction effect (*F*_(10, 5482)_ = 7.616, *p* < 0.001), but no visit-by-group effect, such that, post-season, COLL groups demonstrated longer latency on overlap (High Dose, 2.21% increase; Low Dose, 4.85% increase) and, to a lesser extent, no gap trials (High Dose, 1.97% increase; Low Dose, 1.80% increase), but shorter latency on gap trials (High Dose, 6.84% decrease; Low Dose, 1.26% decrease), relative to pre-season performance. NON-COLL, however, demonstrated relative stability over time (percent change, ≤1.69% across conditions); see Figure 1. There was also a visit-by-group interaction effect on peak velocity (*F*_(2, 2014)_ = 5.208, *p* = 0.006), in which NON-COLL and COLL-Low Dose demonstrated faster velocity at post-season by 2.61% and 2.88%, respectively, but COLL-High Dose demonstrated slower velocity at post-season by 1.58% relative to pre-season performance. There were no visit-by-group effects on prosaccade gain or duration.

#### Antisaccade task

**FIG. 2.**
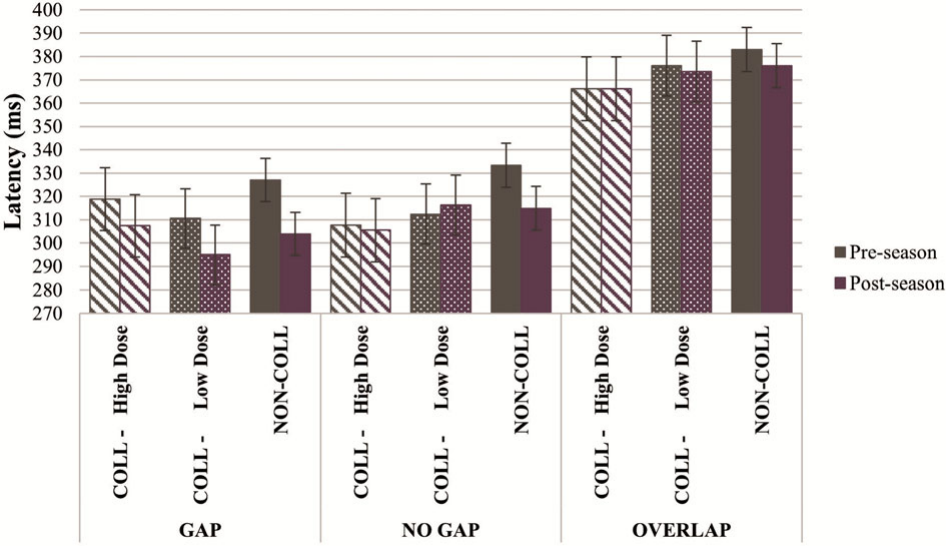
Antisaccade latency by visit, group, and condition. Compared to pre-season performance, *NON-COLL* demonstrated shorter latency at post-season across conditions, whereas *COLL* groups demonstrated relatively stable latency from pre- to post-season averaged across conditions, with greater change observed on gap versus no gap and overlap conditions (visit-by-group-by-condition interaction effect, *p* < 0.001; visit-by-group effect, *p* = 0.001). COLL, collision; NON-COLL, non-collision.

There were significant visit-by-group-by-condition (*F*_(10, 4599)_ = 3.970, *p* < 0.001) and visit-by-group (*F*_(2, 4599)_ = 6.645, *p* = 0.001) interaction effects on antisaccade latency. COLL-High Dose demonstrated stable latency (0.02% decrease, averaged across conditions; *d_ppc2_* = 0.231), COLL-Low Dose demonstrated intermediary shortened latency (2.03% decrease, averaged across conditions; *d_ppc2_* = 0.134), and NON-COLL demonstrated more-pronounced shortened latency (4.33% decrease, averaged across conditions), at post-season relative to pre-season; greater improvement on latency across groups was observed on the gap condition; see Figure 2. There were no visit-by-group effects on antisaccade error rate.

#### Memory-guided saccade task

**FIG. 3.**
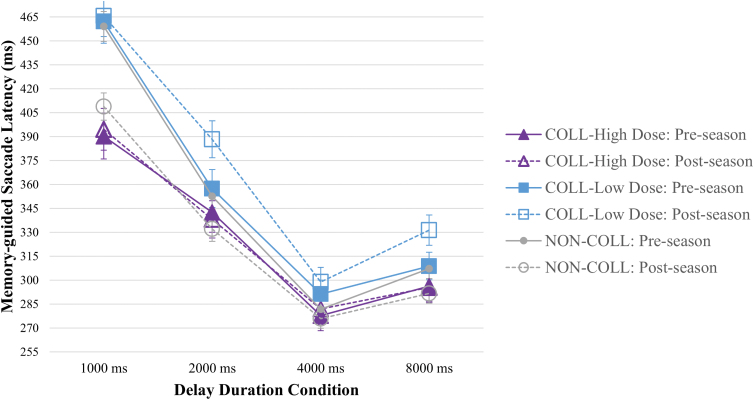
Memory-guided saccade latency by visit, group, and condition. Compared to pre-season performance, *NON-COLL* demonstrated shorter latency across conditions, particularly on 1000-ms delay duration trials, *COLL-High Dose* demonstrated relatively stable latency, and *COLL-Low Dose* demonstrated longer latency, particularly on 2000- and 8000-ms delay duration conditions, at post-season (visit-by-group-by-condition and visit-by-group interaction effects, *p* < 0.001). COLL, collision; NON-COLL, non-collision.

There were significant visit-by-group-by-condition (*F*_(15, 3893)_ = 3.508, *p* < .001) and visit-by-group (*F*_(2, 3893)_ = 20.614, *p* < 0.001) interaction effects on MGS latency, in which NON-COLL demonstrated shorter latency at post-season, relative to pre-season (6.48% decrease averaged across conditions), particularly on 1000-, 2000-, and 8000-ms delay conditions (≥5% decrease); COLL-High Dose demonstrated relatively stable latency from pre- to post-season (0.31% mean increase, averaged across conditions; *d_ppc2_* = 0.293, with change on each condition ≤1.03%); COLL-Low Dose demonstrated longer latency from pre- to post-season (3.86% increase averaged across conditions; *d_ppc2_* = 0.452), particularly on 2000- (8.45% increase) and 8000-ms (6.76% increase) conditions; see Figure 3.

**FIG. 4.**
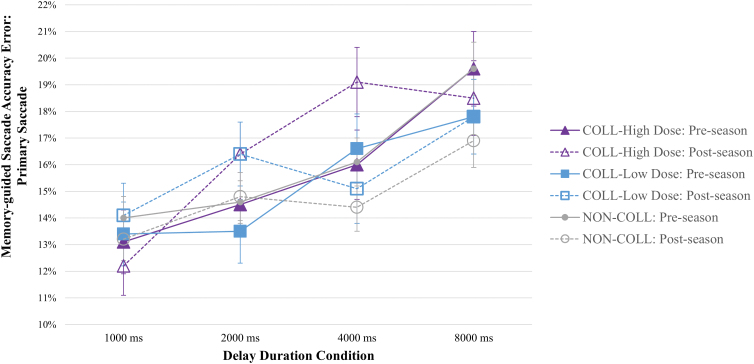
Memory-guided saccade primary saccade accuracy by visit, group, and condition. Compared to pre-season performance, *NON-COLL* demonstrated decreased spatial error, and *COLL* (both groups) demonstrated increased spatial error on the primary saccade (visit-by-group interaction effects, *p* < 0.05; no visit-by-group-by-condition interaction effects). COLL, collision; NON-COLL, non-collision.

There were also significant (uncorrected) visit-by-group interaction effects on the accuracy of the primary saccade (*F*_(2, 3959)_ = 3.061, *p* = 0.047) and accuracy of the resting fixation position (*F*_(5, 4047)_ = 2.397, *p* = 0.035), although there were no visit-by-group-by-condition effects, and these comparisons did not survive Bonferroni's correction. NON-COLL demonstrated improved accuracy of the primary saccade (7.33% decreased error) and the rest position (7.04% decreased error) pre- to post-season, whereas COLL-High Dose and COLL-Low Dose demonstrated decreased accuracy of the primary saccade (3.17% and 3.28% increased error, respectively) and stable accuracy of the rest position over time; see Figure 4. There were no significant visit-by-group-by-condition or visit-by-group effects on delay period errors (i.e., looks to target occurring during the delay period).

### Immediate Post-Concussion Assessment and Cognitive Test

One NON-COLL athlete from the final eye-movement sample did not attend post-season ImPACT testing and was lost to follow-up (ImPACT NON-COLL, *n* = 16). There was no group-by-time interaction effect on ImPACT composite scores (*F*_(8, 56)_ = 0.343, *p* = 0.945); there was a significant effect of time (*F*_(4, 27)_ = 3.524, *p* = 0.019), driven by visual motor composite score increase (improved performance), at post-season relative to pre-season across all groups (*p* < 0.01); see Table 4.

**Table 4.** ImPACT Composite Data by Group and Visit

		Group		
		Collision-High Dose	Collision-Low Dose	Non-collision
		Hockey (*n* = 8)	Rugby (*n* = 9)	(*n* = 16)
Pre-season	Verbal memory	94.00 (4.07)	91.56 (7.52)	90.88 (7.46)
	Visual memory	93.50 (5.18)	83.78 (13.84)	84.19 (8.54)
	Visual motor	48.48 (3.88)	42.08 (6.04)	46.36 (5.47)
	Reaction time	0.57 (0.09)	0.56 (0.06)	0.55 (0.07)
Post-season	Verbal memory	95.38 (7.17)	92.56 (8.92)	90.44 (7.16)
	Visual memory	95.25 (3.62)	87.00 (10.61)	87.94 (6.89)
	Visual motor	49.76 (3.24)	44.15 (5.25	47.99 (3.46)
	Reaction time	0.54 (0.05)	0.55 (0.05)	0.54 (0.05)

Data presented as mean (standard deviation). Verbal and visual memory composite scores represent the average percentage correct across subtests; visual motor composite score represents a summary score of processing speed performance across subtests; reaction time composite score represents the average reaction time (in seconds) for correct responses across subtests. No significant group-by-time interaction effect was detected.

ImPACT, Immediate Post-Concussion Assessment and Cognitive Test.

## Discussion

This study evaluated the use of eye-movement testing to detect change related to participation in club collision sports among male collegiate athletes. We found differential change among COLL versus NON-COLL players from pre- to post-season on several eye-movement measures, namely, speeded aspects of reflexive (prosaccade reaction time and velocity) and executive control and working memory (antisaccade and MGS reaction times) tasks. Broadly speaking, across several reaction-time measures, the COLL-High Dose group did not demonstrate the practice effects that were observed among NON-COLL and, to a lesser extent, COLL-Low Dose athletes. By contrast, no significant differential change among groups was observed on ImPACT—the cognitive measure typically used in the literature to assess cognitive effects of subconcussive hit exposure—from pre- to post-season.

We hypothesized that relative to NON-COLL, COLL athletes would demonstrate longer antisaccade and MGS latencies, whereas NON-COLL would remain stable over time. The direction of our hypotheses were partially supported, in that NON-COLL demonstrated shorter latency at post-season, relative to pre-season, on antisaccade and MGS tasks by 4.33% and 6.48%, reflecting possible practice effects in response latency, whereas COLL demonstrated longer, stable, or more attenuated (relative to NON-COLL) shortened latencies over time. Specifically, COLL-High Dose demonstrated stable antisaccade and MGS latencies from pre- to post-season. By contrast, the COLL-Low Dose group demonstrated 4.65% longer MGS latency and 2.03% shorter antisaccade latency from pre- to post-season. It is surprising that the COLL-Low Dose group demonstrated more adverse change on MGS latency across time relative to the COLL-High Dose group; this may be attributable to small group sizes and intersubject variability. Alternatively, the differential effect may be attributable to a suspected higher magnitude of hits among rugby players because of the lack of protective helmets.

In sum, it appears that the COLL groups demonstrated an attenuated practice effect, relative to NON-COLL, on reaction time on eye-movement saccade tasks with executive control demands. Differential change from pre- to post-season among collision COLL versus NON-COLL groups is consistent with the post-concussion literature, in that past studies have demonstrated longer antisaccade latency among participants with recent concussion versus controls.^28,54–56^ Further, results from this study are similar to those of a parallel longitudinal study of female soccer players, which showed that those with higher estimated exposure to head impacts demonstrated relative stability over time whereas those with lower estimated head injury exposure and non-contact controls showed faster reaction times at post-season relative to pre-season on antisaccade and MGS tasks.^30^

Unlike eye-movement testing results, there were no group-by-time effects observed on ImPACT testing in general or on the ImPACT reaction time or visual motor composite scores. Qualitatively, ImPACT composite scores improved from pre- to post-season on memory and visual motor composites among COLL groups and remained stable or improved more modestly among NON-COLL. This suggests the increased sensitivity of executive saccade tasks, relative to ImPACT testing, to changes resulting from participation in collision versus non-collision sports, presumably as a consequence of subconcussive hit exposure.

This study further evaluated whether, relative to NON-COLL, COLL athletes would demonstrate increased antisaccade error rate and poorer spatial accuracy on the MGS task at post- versus pre-season. Our results did not reveal group-by-time effects on antisaccade error rate, but did yield significant findings for MGS accuracy, though these findings were not significant after correction for multiple comparisons. Specifically, NON-COLL demonstrated improved primary saccade and resting fixation MGS spatial accuracy, whereas COLL (both groups) demonstrated poorer primary saccade accuracy and stable resting fixation accuracy, at post-season relative to pre-season.

With regard to the reflexive prosaccade task, there were significant group-by-time effects on latency and velocity. Specifically, relative to pre-season performance, NON-COLL demonstrated stable latency at post-season. By contrast, COLL groups demonstrated longer latency on overlap and no gap trials, and shorter latency on gap trials, at post- versus pre-season. The prolonged latency on overlap and no gap trials at post-season relative to pre-season among COLL, compared to stable latency among NON-COLL, suggests change as a result of exposure to collision sport participation on reflexive saccade reaction time. In addition, compared to pre-season performance, NON-COLL and COLL-Low Dose demonstrated faster velocity (2.61% and 2.88% increase, respectively) on the prosaccade task, whereas COLL-High Dose demonstrated mildly slower velocity (1.58% decrease), at post-season. This implies a potential subtle dose effect, in which athletes with greater relative exposure to subconcussive hits through increased estimated frequency of participation in collision sport competition demonstrate adverse change on prosaccade velocity performance relative to those with lower relative frequency in engagement, or no engagement at all (i.e., NON-COLL), in collision sport competition.

There are several limitations to this study that warrant mention. Although competition attendance data among COLL athletes suggest a disparate potential exposure to subconcussive hits among hockey (High Dose) versus rugby (Low Dose), we do not have individual, quantitative head injury data to conclusively support that differential change among these groups from pre- to post-season is attributable to variable exposure to head injury. Further, differential change among these groups from pre- to post-season may be attributable to differential, though not statistically significant, time since most recent competition and, by extension, rest and recovery from exposure to subconcussive impacts, given that rugby athletes (Low Dose) were assessed an average of 19.00 days post-competition (SD = 5.07), whereas the hockey athletes (High Dose) were assessed closer to the most recent competition (M = 11.38, SD = 14.00). Future studies should include larger sample sizes, quantitatively monitor head injury exposures for each participant to confirm the sensitivity of eye-movement testing to subconcussive-hit dose effects, and conduct follow-up testing at regular intervals to evaluate the extent to which performance may normalize between collision versus non-collision groups after a period of rest from subconcussive impact exposure among collision athletes.

## Conclusion

In summary, compared to pre-season performance, NON-COLL demonstrated stable, whereas COLL groups demonstrated longer (slower), prosaccade latency on no gap and overlap trials. NON-COLL demonstrated shorter (faster), whereas COLL groups showed stable, longer, or more attenuated improvements in antisaccade and MGS latencies at post-season relative to pre-season. On the MGS task, NON-COLL demonstrated improved spatial accuracy, whereas COLL groups showed poorer primary saccade accuracy and stable resting position accuracy at post-season relative to pre-season (though these findings did not survive Bonferroni's correction for multiple comparisons). Finally, whereas NON-COLL and COLL-Low Dose demonstrated faster post-season relative to pre-season prosaccade velocity, COLL-High Dose showed mildly slower post-season velocity. Overall, there were mild dose effects in prosaccade velocity (greater adverse change among High vs. Low Dose COLL) and antisaccade latency (greater improvement among Low vs. High Dose COLL), but not in other eye-movement measures.

In general, our data demonstrated that male athletes exposed to estimated higher doses of subconcussive impacts did not demonstrate the practice effects on eye-movement testing that were observed among non-collision athletes and, to a lesser extent, collision sport athletes exposed to relatively fewer estimated subconcussive head injury. Whereas the aforementioned effects were statistically significant, the magnitude of effects was generally small, which is not surprising given that the collision athletes did not report increased head injury-related symptoms from pre- to post-season. Additionally, no differential change was observed from pre- to post-season on ImPACT performance among groups, and qualitative review of data demonstrated an unexpected direction of change in which COLL groups improve at post-season to a greater degree than NON-COLL groups on memory and visual motor composite scores. This suggests that eye-movement testing has greater sensitivity to change associated with collision sport participation versus ImPACT. Finally, this study provides convergent data regarding the utility of eye-movement testing in detecting subtle cognitive and sensorimotor change associated with collision sport participation, now demonstrated among both male and female athletes.^30^

## Supplementary Material

Supplemental data

## Abbreviations Used

BDI-II: Beck Depression Inventory-II
COLL: collision
ImPACT: Immediate Post-Concussion Assessment and Cognitive Test
MGS: memory-guided saccade
NON-COLL: non-collision collegiate athletes
PCSS: Post-Concussion Symptom Scale
PSS: Perceived Stress Scale
SD: standard deviation
STAI: State-Trait Anxiety Inventory

## References

[1] Mainwaring, L., Ferdinand Pennock, K.M., Mylabathula, S., and Alavie, B.Z. (2018). Subconcussive head impacts in sport: a systematic review of the evidence. Int. J. Psychophysiol. 132, Pt. A, 39–54. 29402530 10.1016/j.ijpsycho.2018.01.007

[2] McCrory, P., Meeuwisse, W., Dvorak, J., et al. (2017). Consensus statement on concussion in sport—the 5th International Conference on Concussion in Sport held in Berlin, October 2016. Br. J. Sports Med. 51, 838–847. 28446457 10.1136/bjsports-2017-097699

[3] Asken, B.M., Sullan, M.J., DeKosky, S.T., Jaffee, M.S., and Bauer, R.M. (2017). Research gaps and controversies in chronic traumatic encephalopathy: a review. JAMA Neurol. 74, 1255–1262. 28975240 10.1001/jamaneurol.2017.2396

[4] Baugh, C.M., Stamm, J.M., Riley, D.O., Gavett, B.E., Shenton, M.E., Lin, A., Nowinski, C.J., Cantu, R.C., McKee, A.C., and Stern, R.A. (2012). Chronic traumatic encephalopathy: Neurodegeneration following repetitive concussive and subconcussive brain trauma. Brain Imaging and Behav. 6, 244–254. 10.1007/s11682-012-9164-5 22552850

[5] McKee, A.C., Alosco, M.L., and Huber, B.R. (2016). Repetitive head impacts and chronic traumatic encephalopathy. Neurosurg. Clin. N. Am. 27, 529–535. 27637402 10.1016/j.nec.2016.05.009 PMC5028120

[6] Randolph, C. (2014). Is chronic traumatic encephalopathy a real disease? Curr. Sports Med. Rep. 13, 33–37. 24412888 10.1097/OPX.0000000000000170

[7] Hart, J., Kraut, M.A., Womack, K.B., Strain, J., Didehbani, N., Bartz, E., Conover, H., Mansinghani, S., Lu, H., and Cullum, C.M. (2013). Neuroimaging of cognitive dysfunction and depression in aging retired NFL Players: a cross-sectional study. JAMA Neurol. 70, 326–335. 10.1001/2013.jamaneurol.340 23303193 PMC4016798

[8] McKee, A.C., Cantu, R.C., Nowinski, C.J., Hedley-Whyte, E.T., Gavett, B.E., Budson, A.E., Santini, V.E., Lee, H.-S., Kubilus, C.A., and Stern, R.A. (2009). Chronic traumatic encephalopathy in athletes: Progressive tauopathy after repetitive head injury. J. Neuropathol. Exp. Neurol. 68, 709–735. 10.1097/NEN.0b013e3181a9d503 19535999 PMC2945234

[9] Montenigro, P.H., Alosco, M.L., Martin, B.M., Daneshvar, D.H., Mez, J., Chaisson, C.E., Nowinski, C.J., Au, R., McKee, A.C., Cantu, R.C., McClean, M.D., Stern, R.A., and Tripodis, Y. (2017). Cumulative head impact exposure predicts later-life depression, apathy, executive dysfunction, and cognitive impairment in former high school and college football players. J. Neurotrauma 34, 328–340. 10.1089/neu.2016.4413 27029716 PMC5220530

[10] Sowell, E.R., Thompson, P.M., Tessner, K.D., and Toga, A.W. (2001). Mapping continued brain growth and gray matter density reduction in dorsal frontal cortex: inverse relationships during postadolescent brain maturation. J. Neurosci. 21, 8819–8829. 11698594 10.1523/JNEUROSCI.21-22-08819.2001 PMC6762261

[11] Ostby, Y., Tamnes, C.K., Fjell, A.M., Westlye, L.T., Due-Tønnessen, P., and Walhovd, K.B. (2009). Heterogeneity in subcortical brain development: a structural magnetic resonance imaging study of brain maturation from 8 to 30 years. J. Neurosci. 29, 11772–11782. 19776264 10.1523/JNEUROSCI.1242-09.2009 PMC6666647

[12] Bari, S., Svaldi, D.O., Jang, I., Shenk, T.E., Poole, V.N., Lee, T., Dydak, U., Rispoli, J.V., Nauman, E.A., and Talavage, T.M. (2018). Dependence on subconcussive impacts of brain metabolism in collision sport athletes: An MR spectroscopic study. Brain Imaging Behav. 13, 735–739. 10.1007/s11682-018-9861-9 29802602

[13] Bazarian, J.J., Zhu, T., Zhong, J., Janigro, D., Rozen, E., Roberts, Javien, H., Merchant-Borna, K., Abar, B., and Blackman, E.G. (2014). Persistent, long-term cerebral white matter changes after sports-related repetitive head impacts. PLoS One 9, e94734. 24740265 10.1371/journal.pone.0094734 PMC3989251

[14] Chun IY, Mao X, Breedlove EL, Leverenz LJ, Nauman EA, and Talavage, T.M. (2015). DTI Detection of longitudinal WM abnormalities due to accumulated head impacts. Dev. Neuropsychol. 40, 92–97. 25961592 10.1080/87565641.2015.1020945

[15] Davenport, E.M., Whitlow, C.T., Urban, J.E., Espeland, M.A., Jung, Y., Rosenbaum, D.A., Gioia, G.A., Powers, A.K., Stitzel, J.D., and Maldjian, J.A. (2014). Abnormal white matter integrity related to head impact exposure in a season of high school varsity football. J. Neurotrauma 31, 1617–1624. 10.1089/neu.2013.3233 24786802 PMC4170811

[16] Merchant-Borna, K., Asselin, P., Narayan, D., Abar, B., Jones, C.M.C., and Bazarian, J.J. (2016). Novel method of weighting cumulative helmet impacts improves correlation with brain white matter changes after one football season of sub-concussive head blows. Ann. Biomed. Eng. 44, 3679–3692. 27350072 10.1007/s10439-016-1680-9

[17] Shenk, T.E., Robinson, M.E., Svaldi, D.O., Abbas, K., Breedlove, K.M., Leverenz, L.J., Nauman, E.A., and Talavage, T.M. (2015). FMRI of visual working memory in high school football players. Dev. Neuropsychol. 40, 63–68. 10.1080/87565641.2015.1014088 25961587

[18] Sollmann, N., Echlin, P.S., Schultz, V., Viher, P.V., Lyall, A. E., Tripodis, Y., Kaufmann, D., Hartl, E., Kinzel, P., Forwell, L.A., Johnson, A.M., Skopelja, E.N., Lepage, C., Bouix, S., Pasternak, O., Lin, A.P., Shenton, M.E., and Koerte, I.K. (2018). Sex differences in white matter alterations following repetitive subconcussive head impacts in collegiate ice hockey players. NeuroImage Clin. 17, 642–649. 10.1016/j.nicl.2017.11.020 29204342 PMC5709295

[19] Talavage, T.M., Nauman, E.A., Breedlove, E.L., Yoruk, U., Dye, A.E., Morigaki, K.E., Feuer, H., and Leverenz, L.J. (2014). Functionally-detected cognitive impairment in high school football players without clinically-diagnosed concussion. J. Neurotrauma 31, 327–338. 10.1089/neu.2010.1512 20883154 PMC3922228

[20] McAllister, T.W., Flashman, L.A., Maerlender, A., Greenwald, R.M., Beckwith, J.G., Tosteson, T.D., Crisco, J.J., Brolinson, P.G., Duma, S.M., Duhaime, A.-C., Grove, M.R., and Turco, J.H. (2012). Cognitive effects of one season of head impacts in a cohort of collegiate contact sport athletes. Neurology 78, 1777–1784. 10.1212/WNL.0b013e3182582fe7 22592370 PMC3359587

[21] Straume-Naesheim, T.M., Andersen, T.E., Holme, I.M.K., McIntosh, A.S., Dvorak, J., and Bahr R. (2009). Do minor head impacts in soccer cause concussive injury? A prospective case-control study. Neurosurgery 64, 719–725; discussion, 725. 19349829 10.1227/01.NEU.0000340681.12949.6D

[22] Ravdin, L.D., Barr, W.B., Jordan, B., Lathan, W.E., and Relkin, N.R. (2003). Assessment of cognitive recovery following sports related head trauma in boxers. Clin. J. Sport Med. 13, 21–27. 12544160 10.1097/00042752-200301000-00005

[23] Chrisman, S.P.D., Mac Donald, C.L., Friedman, S., Andre, J., Rowhani-Rahbar, A., Drescher, S., Stein, E., Holm, M., Evans, N., Poliakov, A.V., Ching, R.P., Schwien, C.C., Vavilala, M.S., and Rivara, F.P. (2016). Head impact exposure during a weekend youth soccer tournament. J. Child Neurol. 31, 971–978. 10.1177/0883073816634857 26951540

[24] Munce, T.A., Dorman, J.C., Odney, T.O., Thompson, P.A., Valentine, V.D., and Bergeron, M.F. (2014). Effects of youth football on selected clinical measures of neurologic function: a pilot study. J. Child Neurol. 29, 1601–1607. 24272520 10.1177/0883073813509887

[25] Gysland, S.M., Mihalik, J.P., Register-Mihalik, J.K., Trulock, S.C., Shields, E.W., and Guskiewicz, K.M. (2012). The relationship between subconcussive impacts and concussion history on clinical measures of neurologic function in collegiate football players. Ann. Biomed. Eng. 40, 14–22. 21994067 10.1007/s10439-011-0421-3

[26] Miller, J.R., Adamson, G.J., Pink, M.M., and Sweet, J.C. (2007). Comparison of preseason, midseason, and postseason neurocognitive scores in uninjured collegiate football players. Am. J. Sports Med. 35, 1284–1288. 17405886 10.1177/0363546507300261

[27] Cifu, D.X., Wares, J.R., Hoke, K.W., Wetzel, P.A., Gitchel, G., and Carne, W. (2015). Differential eye movements in mild traumatic brain injury versus normal controls. J. Head Trauma Rehabil. 30, 21–28. 24695263 10.1097/HTR.0000000000000036

[28] Heitger, M., Anderson, T.J., Jones, R.D., Dalrymple-Alford, J.C., Frampton, C.M., and Ardagh, M.W. (2004). Eye movement and visuomotor arm movement deficits following mild closed head injury. Brain 127, 575–590. 14736751 10.1093/brain/awh066

[29] Johnson B, Zhang K, Hallett M, Slobounov S. (2015). Functional neuroimaging of acute oculomotor deficits in concussed athletes. Brain Imaging Behav 9, 564–573. 25179246 10.1007/s11682-014-9316-x

[30] Gallagher, V.T., Murthy, P., Stocks, J., Vesci, B., Colegrove, D., Mjaanes, J., Chen, Y., Breiter, H., LaBella, C., Herrold, A.A., and Reilly, J.L. (2020). Differential change in oculomotor performance among female collegiate soccer players versus non-contact athletes from pre- to post-season. Neurotrauma Rep. 1, 169–180. 10.1089/neur.2020.0051 33274345 PMC7703496

[31] Heitger, M.H., Jones, R.D., Macleod, A.D., Snell, D.L., Frampton, C.M., and Anderson, T.J. (2009). Impaired eye movements in post-concussion syndrome indicate suboptimal brain function beyond the influence of depression, malingering or intellectual ability. Brain 132, 2850–2870. 19617197 10.1093/brain/awp181

[32] Leigh, R.J., and Kennard, C. (2004). Using saccades as a research tool in the clinical neurosciences. Brain 127, 460–477. 14607787 10.1093/brain/awh035

[33] Brainard, L.L., Beckwith, J.G., Chu, J.J., Crisco, J.J., McAllister, T.W., Duhaime, A.-C., Maerlender, A.C., and Greenwald, R. M. (2012). Gender differences in head impacts sustained by collegiate ice hockey players. Med. Sci. Sports Exerc. 44, 297–304. 10.1249/MSS.0b013e31822b0ab4 21716150 PMC3694342

[34] King, D., Hume, P.A., Brughelli, M., and Gissane, C. (2015). Instrumented mouthguard acceleration analyses for head impacts in amateur rugby union players over a season of matches. Am. J. Sports Med. 43, 614–624. 25535096 10.1177/0363546514560876

[35] Wilcox, B.J., Beckwith, J.G., Greenwald, R.M., Chu, J.J., McAllister, T.W., Flashman, L.A., Maerlender, A.C., Duhaime, A.-C., and Crisco, J.J. (2014). Head impact exposure in male and female collegiate ice hockey players. J. Biomech. 47, 109–114. 10.1016/j.jbiomech.2013.10.004 24210478 PMC3902856

[36] Belanger, H.G., Vanderploeg, R.D., and McAllister, T. (2016). Subconcussive blows to the head: a formative review of short-term clinical outcomes. J. Head Trauma Rehabil. 31, 159–166. 25931186 10.1097/HTR.0000000000000138

[37] Reilly, J.L., Harris, M.S.H., Khine, T.T., Keshavan, M.S., and Sweeney, J.A. (2008). Reduced attentional engagement contributes to deficits in prefrontal inhibitory control in schizophrenia. Biol. Psychiatry 63, 776–783. 18191110 10.1016/j.biopsych.2007.11.009 PMC2366792

[38] Reilly, J.L., Frankovich, K., Hill, S., Gershon, E.S., Keefe, R.S.E., Keshavan, M.S., Pearlson, G.D., Tamminga, C.A., and Sweeney, J.A. (2014). Elevated antisaccade error rate as an intermediate phenotype for psychosis across diagnostic categories. Schizophr. Bull. 40, 1011–1021. 10.1093/schbul/sbt132 24080895 PMC4133662

[39] Lunn, J., Donovan, T., Litchfield, D., Lewis, C., Davies, R., and Crawford, T. (2016). Saccadic eye movement abnormalities in children with epilepsy. PLoS One 11, e0160508. 27483011 10.1371/journal.pone.0160508 PMC4970731

[40] Mani, R., Asper, L., and Khuu, S.K. (2018). Deficits in saccades and smooth-pursuit eye movements in adults with traumatic brain injury: a systematic review and meta-analysis. Brain Inj. 32, 1315–1336. 29913089 10.1080/02699052.2018.1483030

[41] Reilly, J.L., Lencer, R., Bishop, J.R., Keedy, S., and Sweeney, J.A. (2008). Pharmacological treatment effects on eye movement control. Brain Cogn. 68, 415–435. 19028266 10.1016/j.bandc.2008.08.026 PMC3159189

[42] Iverson, G.L., Lovell, M.R., and Collins, M.W. (2003). Interpreting change on ImPACT following sport concussion. Clin. Neuropsychol. 17, 460–467. 15168911 10.1076/clin.17.4.460.27934

[43] Pedersen, E.R., Grow, J., Duncan, S., Neighbors, C., and Larimer, M.E. (2012). Concurrent validity of an online version of the Timeline Followback assessment. Psychol. Addict. Behav. 26, 672–677. 22486334 10.1037/a0027945 PMC3714014

[44] Robinson, S.M., Sobell, L.C., Sobell, M.B., and Leo, G.I. (2014). Reliability of the Timeline Followback for cocaine, cannabis, and cigarette use. Psychol. Addict. Behav. 28, 154–162. 23276315 10.1037/a0030992

[45] Sobell, L.C., Agrawal, S., Sobell, M.B., Leo, G.I., Young, L.J., Cunningham, J.A., and Simco, E.R. (2003). Comparison of a quick drinking screen with the timeline followback for individuals with alcohol problems. J. Stud. Alcohol 64, 858–861. 10.15288/jsa.2003.64.858 14743950

[46] Hollingshead, A.B. (1975). Four Factor Index of Social Status. Yale University: New Haven, CT.

[47] Lovell, M.R., Iverson, G.L., Collins, M.W., Podell, K., Johnston, K.M., Pardini, D., Pardini, J., Norwig, J., and Maroon, J.C. (2006). Measurement of symptoms following sports-related concussion: reliability and normative data for the post-concussion scale. Appl. Neuropsychol. 13, 166–174. 10.1207/s15324826an1303_4 17361669

[48] Beck, A.T., Steer, R.A., Ball, R., and Ranieri, W.F. (1996). Comparison of Beck Depression Inventories-IA and-II in psychiatric outpatients. J. Pers. Assess. 67, 588–597. 8991972 10.1207/s15327752jpa6703_13

[49] Sprinkle, S.D., Lurie, D., Insko, S.L., Atkinson, G., Jones, G.L., Logan, A.R., and Bissada, N.N. (2002). Criterion validity, severity cut scores, and test-retest reliability of the beck depression inventory-II in a university counseling center sample. J. Couns. Psychol. 49, 381–385.

[50] Spielberger, C.D. (2010). State-Trait Anxiety Inventory, in: *The Corsini Encyclopedia of Psychology*. I.B. Weiner and W.E. Craighead (eds). John Wiley & Sons: Hoboken, NJ.

[51] Cohen, S., Kamarck, T., and Mermelstein, R. (1983). A global measure of perceived stress. J. Health Soc. Behav. 24, 385–396. 6668417

[52] Lee, E.-H. (2012). Review of the psychometric evidence of the Perceived Stress Scale. Asian Nurs. Res. 6, 121–127. 10.1016/j.anr.2012.08.004 25031113

[53] IBM Corp. (2017). IBM SPSS Statistics for Windows. IBM Corp.: Armonk, NY.

[54] Morris, S.B. (2008). Estimating effect sizes from pretest-posttest-control group designs. Organ. Res. Methods 11, 364–386.

[55] Landry, A.P., Ting, W.K.C., Zador, Z., Sadeghian, A., and Cusimano, M.D. (2018). Using artificial neural networks to identify patients with concussion and postconcussion syndrome based on antisaccades. J. Neurosurg. doi: 10.3171/2018.6.JNS18607. 30497186

[56] Ting, W.K.C., Schweizer, T.A., Topolovec-Vranic, J., and Cusimano, M.D. (2015). Antisaccadic eye movements are correlated with corpus callosum white matter mean diffusivity, stroop performance, and symptom burden in mild traumatic brain injury and concussion. Front. Neurol. 6, 271. 26834693 10.3389/fneur.2015.00271 PMC4716139

[57] Webb, B., Humphreys, D., and Heath, M. (2018). Oculomotor executive dysfunction during the early and later stages of sport-related concussion recovery. J. Neurotrauma 35, 1874–1881. 30074868 10.1089/neu.2018.5673

