# Supplementary material for: Eye Movements Detect Differential Change after Participation in Male Collegiate Collision versus Non-Collision Sports
Source: Neurotrauma Rep. 2021 Oct 7;2(1):440–52. doi: 10.1089/neur.2021.0030 (PMC8655805; doi:10.1089/neur.2021.0030)
Supplement: Supplemental data [file Suppl-TableS1-S3.docx]

Supplementary Tables

| Supplementary Table 1  *Prosaccade Task: Primary Saccade Mean and Standard Deviation Data by Group, Condition, and Visit* | | | | | | | | | |
| --- | --- | --- | --- | --- | --- | --- | --- | --- | --- |
|  |  | *COLL-High Dose* (*n* = 8) | | | | | | | |
|  |  | GAP | | NO GAP | | OVERLAP | | OVERALL | |
|  |  | M | SD | M | SD | M | SD | M | SD |
| Pre-season | Latency (ms) | 173.47 | 35.56 | 175.73 | 27.83 | 227.18 | 57.02 | *192.13* | *40.14* |
|  | Accuracy^a^ | 0.98 | 0.07 | 0.97 | 0.07 | 0.99 | 0.07 | *0.98* | *0.07* |
|  | Gain^b^ | 1.02 | 0.08 | 1.01 | 0.08 | 1.02 | 0.07 | *1.02* | *0.08* |
|  | Duration (ms) | 58.93 | 8.36 | 58.06 | 8.73 | 59.54 | 9.58 | *58.84* | *8.89* |
|  | Peak Velocity (deg/sec) | 391.16 | 65.90 | 396.07 | 64.00 | 390.52 | 62.49 | *392.58* | *64.13* |
| Post-season | Latency (ms) | 161.61 | 34.08 | 179.20 | 26.34 | 232.21 | 58.87 | *191.01* | *39.76* |
|  | Accuracy^a^ | 0.97 | 0.08 | 0.97 | 0.07 | 0.98 | 0.07 | *0.97* | *0.07* |
|  | Gain^b^ | 1.01 | 0.08 | 1.01 | 0.08 | 1.02 | 0.08 | *1.01* | *0.08* |
|  | Duration (ms) | 57.30 | 9.00 | 57.67 | 8.80 | 58.59 | 9.24 | *57.85* | *9.01* |
|  | Peak Velocity (deg/sec) | 395.93 | 64.55 | 389.83 | 57.04 | 396.81 | 66.00 | *394.19* | *62.53* |
|  |  | *COLL-Low Dose* (*n* = 9) | | | | | | | |
|  |  | GAP | | NO GAP | | OVERLAP | | OVERALL | |
|  |  | M | SD | M | SD | M | SD | M | SD |
| Pre-season | Latency (ms) | 164.89 | 36.77 | 181.99 | 28.40 | 209.62 | 52.60 | *185.50* | *39.26* |
|  | Accuracy^a^ | 0.98 | 0.08 | 0.98 | 0.08 | 0.97 | 0.07 | *0.98* | *0.08* |
|  | Gain^b^ | 1.01 | 0.08 | 1.02 | 0.07 | 1.01 | 0.08 | *1.01* | *0.08* |
|  | Duration (ms) | 60.22 | 10.17 | 59.69 | 9.50 | 59.43 | 10.65 | *59.78* | *10.11* |
|  | Peak Velocity (deg/sec) | 411.26 | 65.84 | 395.48 | 62.93 | 395.97 | 71.08 | *400.90* | *66.62* |
| Post-season | Latency (ms) | 162.81 | 33.22 | 185.27 | 29.39 | 219.78 | 54.34 | *189.29* | *38.98* |
|  | Accuracy^a^ | 0.98 | 0.08 | 0.98 | 0.07 | 0.99 | 0.07 | *0.98* | *0.07* |
|  | Gain^b^ | 1.02 | 0.08 | 1.01 | 0.07 | 1.03 | 0.08 | *1.02* | *0.08* |
|  | Duration (ms) | 59.54 | 10.01 | 60.42 | 9.81 | 59.48 | 9.86 | *59.81* | *9.89* |
|  | Peak Velocity (deg/sec) | 409.88 | 72.76 | 406.88 | 65.88 | 392.03 | 68.73 | *402.93* | *69.12* |
|  |  | *NON-COLL* (*n* = 17) | | | | | | | |
|  |  | GAP | | NO GAP | | OVERLAP | | OVERALL | |
|  |  | M | SD | M | SD | M | SD | M | SD |
| Pre-season | Latency (ms) | 160.69 | 32.28 | 182.27 | 28.61 | 229.46 | 56.32 | *190.81* | *39.07* |
|  | Accuracy^a^ | 0.98 | 0.08 | 0.98 | 0.08 | 0.99 | 0.07 | *0.98* | *0.08* |
|  | Gain^b^ | 1.01 | 0.08 | 1.01 | 0.08 | 1.02 | 0.08 | *1.01* | *0.08* |
|  | Duration (ms) | 60.14 | 9.86 | 60.32 | 9.53 | 60.75 | 9.76 | *60.40* | *9.72* |
|  | Peak Velocity (deg/sec) | 377.95 | 81.62 | 372.22 | 76.30 | 371.91 | 73.68 | *374.03* | *77.20* |
| Post-season | Latency (ms) | 163.40 | 33.68 | 179.62 | 26.12 | 228.62 | 56.53 | *190.55* | *38.78* |
|  | Accuracy^a^ | 0.99 | 0.07 | 0.99 | 0.07 | 0.99 | 0.07 | *0.99* | *0.07* |
|  | Gain^b^ | 1.02 | 0.08 | 1.02 | 0.07 | 1.03 | 0.07 | *1.02* | *0.07* |
|  | Duration (ms) | 60.01 | 9.72 | 60.58 | 9.50 | 61.33 | 9.65 | *60.64* | *9.62* |
|  | Peak Velocity (deg/sec) | 379.63 | 72.15 | 381.95 | 72.44 | 380.79 | 77.90 | *380.79* | *74.16* |
| ^a^Accuracy represents the ratio of the distance (in pixels) between central fixation and the end location of the primary saccade:the pixel location of the target. ^b^Gain represents the ratio of saccade amplitude:target degrees. | | | | | | | | | |
|  |  |  |  |  |  |  |  |  |  |
|  |  |  |  |  |  |  |  |  |  |

| Supplementary Table 2  *Anti-saccade Task: Primary Saccade Mean and Standard Deviation Data by Group, Condition, and Visit* | | | | | | | | | |
| --- | --- | --- | --- | --- | --- | --- | --- | --- | --- |
|  |  | *COLL-High Dose* (*n* = 8) | | | | | | | |
|  |  | GAP | | NO GAP | | OVERLAP | | OVERALL | |
|  |  | M | SD | M | SD | M | SD | M | SD |
| Pre-season | Latency (ms) | 316.17 | 63.09 | 305.18 | 58.88 | 365.78 | 75.92 | *329.04* | *65.96* |
|  | Accuracy^a^ | 0.84 | 0.22 | 0.83 | 0.25 | 0.84 | 0.23 | *0.84* | *0.23* |
|  | Gain^b^ | 0.88 | 0.23 | 0.87 | 0.25 | 0.88 | 0.23 | *0.88* | *0.24* |
|  | Duration (ms) | 60.47 | 13.51 | 60.99 | 13.27 | 60.27 | 13.03 | *60.58* | *13.27* |
|  | Peak Velocity (deg/sec) | 325.85 | 70.81 | 324.96 | 70.51 | 332.59 | 72.03 | *327.80* | *71.12* |
|  | Error Rate | 0.2 | 0.16 | 0.2 | 0.1 | 0.13 | 0.1 | *0.18* | *0.12* |
| Post-season | Latency (ms) | 310.01 | 60.79 | 309.56 | 60.05 | 367.32 | 76.19 | *328.96* | *65.68* |
|  | Accuracy^a^ | 0.84 | 0.22 | 0.86 | 0.22 | 0.86 | 0.21 | *0.85* | *0.22* |
|  | Gain^b^ | 0.87 | 0.23 | 0.89 | 0.21 | 0.90 | 0.21 | *0.89* | *0.22* |
|  | Duration (ms) | 63.02 | 15.64 | 61.51 | 14.01 | 63.15 | 14.33 | *62.56* | *14.66* |
|  | Peak Velocity (deg/sec) | 304.36 | 65.84 | 316.52 | 62.97 | 314.19 | 62.56 | *311.69* | *63.79* |
|  | Error Rate | 0.14 | 0.11 | 0.2 | 0.15 | 0.09 | 0.11 | *0.14* | *0.12* |
|  |  | *COLL-Low Dose* (*n* = 9) | | | | | | | |
|  |  | GAP | | NO GAP | | OVERLAP | | OVERALL | |
|  |  | M | SD | M | SD | M | SD | M | SD |
| Pre-season | Latency (ms) | 316.07 | 55.97 | 317.88 | 56.93 | 375.96 | 63.79 | *336.64* | *58.90* |
|  | Accuracy^a^ | 0.93 | 0.23 | 0.93 | 0.25 | 0.97 | 0.24 | *0.94* | *0.24* |
|  | Gain^b^ | 0.97 | 0.23 | 0.97 | 0.26 | 1.01 | 0.24 | *0.98* | *0.24* |
|  | Duration (ms) | 66.77 | 16.45 | 67.98 | 17.04 | 68.64 | 15.46 | *67.80* | *16.32* |
|  | Peak Velocity (deg/sec) | 345.54 | 73.90 | 341.61 | 74.57 | 345.69 | 86.38 | *344.28* | *78.28* |
|  | Error Rate | 0.28 | 0.22 | 0.25 | 0.18 | 0.13 | 0.12 | *0.22* | *0.17* |
| Post-season | Latency (ms) | 298.05 | 56.75 | 317.28 | 68.16 | 374.10 | 66.01 | *329.81* | *63.64* |
|  | Accuracy^a^ | 0.93 | 0.23 | 0.94 | 0.23 | 0.94 | 0.22 | *0.94* | *0.23* |
|  | Gain^b^ | 0.96 | 0.24 | 0.97 | 0.23 | 0.98 | 0.22 | *0.97* | *0.23* |
|  | Duration (ms) | 68.71 | 17.16 | 69.94 | 17.23 | 69.78 | 16.14 | *69.48* | *16.84* |
|  | Peak Velocity (deg/sec) | 336.03 | 70.59 | 342.58 | 73.94 | 342.21 | 65.19 | *340.27* | *69.91* |
|  | Error Rate | 0.21 | 0.2 | 0.21 | 0.17 | 0.16 | 0.19 | *0.19* | *0.19* |
|  |  | *NON-COLL* (*n* = 17) | | | | | | | |
|  |  | GAP | | NO GAP | | OVERLAP | | OVERALL | |
|  |  | M | SD | M | SD | M | SD | M | SD |
| Pre-season | Latency (ms) | 323.48 | 57.02 | 332.88 | 64.59 | 382.90 | 68.65 | *346.42* | *63.42* |
|  | Accuracy^a^ | 0.89 | 0.24 | 0.90 | 0.23 | 0.88 | 0.24 | *0.89* | *0.24* |
|  | Gain^b^ | 0.93 | 0.24 | 0.93 | 0.24 | 0.92 | 0.24 | *0.93* | *0.24* |
|  | Duration (ms) | 67.74 | 16.79 | 66.53 | 16.16 | 67.79 | 17.77 | *67.35* | *16.91* |
|  | Peak Velocity (deg/sec) | 297.85 | 84.53 | 299.46 | 85.46 | 298.86 | 84.19 | *298.72* | *84.73* |
|  | Error Rate | 0.20 | 0.14 | 0.25 | 0.13 | 0.16 | 0.11 | *0.20* | *0.13* |
| Post-season | Latency (ms) | 304.24 | 57.38 | 315.12 | 60.06 | 374.86 | 74.51 | *331.41* | *63.98* |
|  | Accuracy^a^ | 0.92 | 0.24 | 0.93 | 0.23 | 0.89 | 0.22 | *0.91* | *0.23* |
|  | Gain^b^ | 0.95 | 0.24 | 0.97 | 0.24 | 0.92 | 0.23 | *0.95* | *0.24* |
|  | Duration (ms) | 67.61 | 16.25 | 68.53 | 15.45 | 66.78 | 15.43 | *67.64* | *15.71* |
|  | Peak Velocity (deg/sec) | 316.22 | 81.40 | 304.72 | 77.68 | 305.46 | 81.97 | *308.80* | *80.35* |
|  | Error Rate | 0.16 | 0.1 | 0.19 | 0.12 | 0.13 | 0.07 | *0.16* | *0.10* |
| ^a^Accuracy represents the ratio of the distance (in pixels) between central fixation and the end location of the primary saccade:the pixel location of the target. ^b^Gain represents the ratio of saccade amplitude:target degrees. | | | | | | | | | |
|  |  |  |  |  |  |  |  |  |  |
|  |  |  |  |  |  |  |  |  |  |

| Supplementary Table 3  *Memory-Guided Saccade Task: Saccade Mean and Standard Deviation Data by Group, Condition, and Visit* | | | | | | | | | | | |
| --- | --- | --- | --- | --- | --- | --- | --- | --- | --- | --- | --- |
|  |  | *COLL-High Dose* (*n* = 8) | | | | | | | | | |
|  |  | 1000 ms | | 2000 ms | | 4000 ms | | 8000 ms | | Overall | |
|  |  | M | SD | M | SD | M | SD | M | SD | M | SD |
| Pre-season | Latency (ms) | 397.45 | 117.14 | 341.64 | 102.85 | 283.13 | 47.09 | 300.59 | 45.44 | *330.70* | *78.13* |
|  | Accuracy Error: Primary Saccade^a^ | 0.13 | 0.11 | 0.14 | 0.11 | 0.16 | 0.12 | 0.20 | 0.14 | *0.16* | *0.12* |
|  | Accuracy Error: Resting Position^b^ | 0.08 | 0.06 | 0.08 | 0.06 | 0.09 | 0.06 | 0.10 | 0.08 | *0.09* | *0.07* |
|  | Delay Error Rate^c^ | 0.13 | 0.14 | 0.12 | 0.12 | 0.19 | 0.20 | 0.17 | 0.13 | *0.15* | *0.15* |
|  | Delay Error Rate: Stimulus Driven Only^d^ | 0.11 | 0.16 | 0.07 | 0.09 | 0.13 | 0.20 | 0.08 | 0.14 | *0.10* | *0.15* |
| Post-season | Latency (ms) | 399.72 | 114.01 | 339.96 | 85.39 | 286.05 | 49.89 | 301.12 | 49.92 | *331.71* | *74.80* |
|  | Accuracy Error: Primary Saccade^a^ | 0.12 | 0.10 | 0.16 | 0.12 | 0.19 | 0.14 | 0.18 | 0.14 | *0.16* | *0.13* |
|  | Accuracy Error: Resting Position^b^ | 0.08 | 0.07 | 0.08 | 0.07 | 0.09 | 0.08 | 0.10 | 0.08 | *0.09* | *0.08* |
|  | Delay Error Rate^c^ | 0.11 | 0.09 | 0.08 | 0.07 | 0.16 | 0.15 | 0.18 | 0.19 | *0.13* | *0.13* |
|  | Delay Error Rate: Stimulus Driven Only^d^ | 0.10 | 0.08 | 0.04 | 0.05 | 0.12 | 0.17 | 0.08 | 0.08 | *0.09* | *0.10* |
|  |  | *COLL-Low Dose* (*n* = 9) | | | | | | | | | |
|  |  | 1000 ms | | 2000 ms | | 4000 ms | | 8000 ms | | Overall | |
|  |  | M | SD | M | SD | M | SD | M | SD | M | SD |
| Pre-season | Latency (ms) | 462.99 | 107.29 | 359.54 | 104.72 | 294.39 | 52.38 | 309.98 | 47.6 | *356.73* | *78.00* |
|  | Accuracy Error: Primary Saccade^a^ | 0.13 | 0.11 | 0.13 | 0.11 | 0.17 | 0.13 | 0.18 | 0.13 | *0.15* | *0.12* |
|  | Accuracy Error: Resting Position^b^ | 0.10 | 0.08 | 0.10 | 0.08 | 0.10 | 0.07 | 0.12 | 0.08 | *0.11* | *0.08* |
|  | Delay Error Rate^c^ | 0.26 | 0.26 | 0.28 | 0.17 | 0.27 | 0.22 | 0.27 | 0.24 | *0.27* | *0.22* |
|  | Delay Error Rate: Stimulus Driven Only^d^ | 0.20 | 0.23 | 0.19 | 0.19 | 0.21 | 0.22 | 0.17 | 0.22 | *0.19* | *0.22* |
| Post-season | Latency (ms) | 465.55 | 113.46 | 389.92 | 105.22 | 295.52 | 53.17 | 330.94 | 50.61 | *370.48* | *80.62* |
|  | Accuracy Error: Primary Saccade^a^ | 0.14 | 0.10 | 0.16 | 0.13 | 0.15 | 0.11 | 0.18 | 0.15 | *0.16* | *0.12* |
|  | Accuracy Error: Resting Position^b^ | 0.10 | 0.08 | 0.10 | 0.08 | 0.10 | 0.07 | 0.11 | 0.08 | *0.10* | *0.08* |
|  | Delay Error Rate^c^ | 0.20 | 0.23 | 0.21 | 0.21 | 0.21 | 0.21 | 0.27 | 0.24 | *0.22* | *0.22* |
|  | Delay Error Rate: Stimulus Driven Only^d^ | 0.18 | 0.24 | 0.16 | 0.19 | 0.14 | 0.16 | 0.17 | 0.14 | *0.16* | *0.18* |
|  |  | *NON-COLL* (*n* = 17) | | | | | | | |  |  |
|  |  | 1000 ms | | 2000 ms | | 4000 ms | | 8000 ms | | Overall | |
|  |  | M | SD | M | SD | M | SD | M | SD | M | SD |
| Pre-season | Latency (ms) | 457.69 | 135.02 | 350.48 | 99.99 | 280.47 | 48.12 | 306.49 | 48.61 | *348.78* | *82.94* |
|  | Accuracy Error: Primary Saccade^a^ | 0.14 | 0.11 | 0.14 | 0.12 | 0.16 | 0.13 | 0.194 | 0.142 | *0.16* | *0.12* |
|  | Accuracy Error: Resting Position^b^ | 0.10 | 0.07 | 0.09 | 0.08 | 0.11 | 0.07 | 0.10 | 0.08 | *0.10* | *0.08* |
|  | Delay Error Rate^c^ | 0.15 | 0.11 | 0.19 | 0.17 | 0.17 | 0.14 | 0.21 | 0.15 | *0.18* | *0.14* |
|  | Delay Error Rate: Stimulus Driven Only^d^ | 0.10 | 0.10 | 0.10 | 0.15 | 0.09 | 0.14 | 0.09 | 0.12 | *0.10* | *0.13* |
| Post-season | Latency (ms) | 407.62 | 110.54 | 331.02 | 95.56 | 275.02 | 44.49 | 291.01 | 48.07 | *326.17* | *74.67* |
|  | Accuracy Error: Primary Saccade^a^ | 0.13 | 0.10 | 0.15 | 0.12 | 0.14 | 0.12 | 0.1686 | 0.137 | *0.15* | *0.12* |
|  | Accuracy Error: Resting Position^b^ | 0.09 | 0.07 | 0.09 | 0.07 | 0.10 | 0.07 | 0.09 | 0.07 | *0.09* | *0.07* |
|  | Delay Error Rate^c^ | 0.14 | 0.13 | 0.16 | 0.13 | 0.20 | 0.14 | 0.17 | 0.15 | *0.17* | *0.14* |
|  | Delay Error Rate: Stimulus Driven Only^d^ | 0.09 | 0.13 | 0.10 | 0.10 | 0.11 | 0.12 | 0.07 | 0.11 | *0.09* | *0.12* |
| ^a^Accuracy Error: Primary Saccade = Absolute value[1-(the distance between central fixation and the end location of the primary saccade/target location )]. ^b^Accuracy Error: Resting Position = Absolute value[1-(the distance between central fixation and the location of the rest position/target location)]. ^c^Delay Error Rate = # of trials with 1+ errors during entire delay duration/total # of trials. ^d^Delay Error Rate: Stimulus Driven Only = # of trials with 1+ errors occurring 120–300ms after target appearance/total # of trials. | | | | | | | | | | | |
|  |  |  |  |  |  |  |  |  |  |  |  |
